# Still unseen and ignored: Tracking community knowledge and attitudes about child abuse and child protection in Australia

**DOI:** 10.3389/fpsyg.2022.860212

**Published:** 2022-09-02

**Authors:** Joseph Tucci, Janise Mitchell

**Affiliations:** Australian Childhood Foundation, Melbourne, VIC, Australia

**Keywords:** community attitude, child abuse, prevention, child fatality, awareness

## Abstract

In September 2003, we released the first results of a national community attitude tracking study about child abuse and child protection. At that time, we concluded that as a community, violence against children was tolerated. The community did not understand or appreciate the seriousness, size and cost of child abuse in Australia. There was evidence that child abuse was not viewed as an important challenge facing children in Australia. A second study conducted in 2006 found that nothing much had changed, indeed community engagement with the issue of child abuse may have even deteriorated. A third study in 2010 found that the community actively avoids the problem of child abuse rating it less concerning than high petrol prices. In 2021, 18 years after the first report was published, we have concluded again that child abuse remains out of sight and out of mind as a community concern. This article describes the findings of this fourth iteration of our survey and analyses the implications for ensuring that individuals are more engaged and committed to taking action to preventing child abuse and/or protecting children from violation.

## Introduction

Arguably, child abuse does not remain in community consciousness for very long. As Durfee and Tilton- Durfee (2013) noted that it took the publication of “The Battered Child Syndrome” by Kempe et al. (1962) to break a 102 year silence that followed the world first 1,860 study about fatal child abuse by French Physician—Ambrose Tardieu.

In his seminal work, Kempe et al. (1962) wrote then that as a clinical condition in young children who experienced serious physical abuse from a caregiver,

*“…it is a significant cause of childhood disability and death….yet there is reluctance on the part of many physicians to accept the radiologic signs as indications of repetitive trauma and possible abuse (p. 143–144).”*

Kempe suggested that

*“…many physicians find it hard to believe that parents could have attacked their children and they attempt to obliterate such suspicions from their mind (p. 146).”*

Four years later in 1966, Robert and John Birrell wrote the first paper to bring to light the extent to which children were being physically abused and neglected in Australia—a proportion of whom were killed as a result. As brothers and both pioneering doctors themselves, they prophetically argued then that

*“…one of the main reasons why the maltreatment syndrome is not well recognised is the general attitude of disbelief and incredulity that people would or could do such things to little children. The attitude is widespread, extending to housewife, doctor, lawyer, and even policeman. The hospital staff…tend often not to think of violence, particularly when faced by a neatly dressed and plausible husband or wife…Recognition of the “Battered Child Syndrome” is naturally the crux of any program of prevention… (Birrell and Birrell, p. 1137).”*

Maintaining that terms missing in professional conceptualization can lead to blind spots in practice, Durfee and Tilton- Durfee (2013) found that child abuse was only formally added to the 1965 edition of Index Medicus—the most comprehensive bibliographic database of life science and biomedical science information of its time. It took another 5 years for the term infanticide—the killing of a child—to be included.

Of course, child abuse is not invisible. It is rendered so through the prevailing attitudes of the community. For this reason, it is critical to not only ascertain what these perceptions actually are, but also to understand if they change over time and how.

In 2003, we began what has become the longest running community tracking research examining the attitudes and perceptions of adult Australians about child abuse and child protection. This paper presents an analysis of the fourth iteration in this series which has been running for almost two decades (Tucci et al., 2001, 2003, 2006, 2010).

It is not difficult to connect the ways that individuals view issues that affect the safety and wellbeing of children with their level of commitment to support efforts to prevent child abuse and protect children from violation. As such, the reduction of violence against children depends significantly on these views.

## Methodology

### Aims

The key objectives of this research were to

• assess the degree to which child abuse is considered a community concern;
• gauge the accuracy of public knowledge about the extent, nature and impact of child abuse; and,
• track community attitudes about the challenges facing children in relation to child abuse and child protection.

### Survey method

An online survey of 1,009 adults aged 18 years and over in Australia was completed in November 2020 by EY Sweeney. A sample of 1,009 yields a high degree of statistical precision, with a margin of error of ± 3.1%. The margin of error is how likely it is that a result will differ from the “true” result (if everyone in Australia was surveyed). A maximum margin of error of ± 3.1 at a 95% confidence level means that for a survey result of a 50%, if the survey was repeated multiple times 95% of these times the survey result will be between 46.9 and 53.1%. Further to this, the data was weighted to the latest available ABS census statistics on state, gender and age to ensure a nationally representative sample.

A sample of telephone interviews was also conducted in order to compare key questions to historical results so as to calibrate the data if required given the shift from predominantly telephone surveying in 2009 to a predominantly online survey in 2020.

The composition and background of the sample are detailed in the Supplementary Tables A1–A5 in Supplementary Appendix A.

## Survey sample

### Critical findings

#### Child abuse remains unseen

In Table 1, the key findings mirror the results from the previous surveys in which unprompted recall for child abuse as a community concern remains low. It has shifted very little over the past 18 years. If anything, it has decreased since its peak in 2006.

**TABLE 1:** Key findings.

Child abuse is rated thirteenth on a list of community issues.
There were six times more people who had no concerns at all than there were people who were concerned about the problem of child abuse.
Child abuse is rated less concerning than transport, traffic, and roads.
Community concern about child abuse has not changed since 2003.
71% of respondents did not recall seeing or hearing any advertising or news related to child abuse or the protection of children in the past 12 months.

As noted in Supplementary Appendix B (Supplementary Table B.1), COVID-19 and related issues have understandably taken over as the primary concerns of adults in the community. It is hardly surprising given its scale and impact. In 2021, Tucci, Mitchell, and Thomas reported the findings of a community survey that demonstrated there is no doubt that COVID-19 has led to an immediate and chronic fallout of negative effects on the mental health and wellbeing of children and parents across Australia. A quarter of parents felt that they were failing their children and more than a third stated that they had lost confidence about their parenting. These problems emerged at exactly the time when parents noticed that their children needed more re-assurance and were experiencing signs of heightened stress such as eating and sleeping disturbances.

A third of parents felt isolated and left without adequate support. Almost 40% were worried that their own stress and mental health was adversely affecting the wellbeing of their children.

Concerningly, almost a third of parents were frightened that the impact of COVID-19 will have lasting mental health impacts for their children such as ongoing heightened anxiety and stress. 1 in 5 parents were concerned about their children’s future social development and self-confidence.

Social distancing restrictions and lockdown measures have resulted in an overwhelming number of children experiencing a range of losses in their daily lives. The absence of their ability to play with friends during lockdown was acutely experienced by 8 out of 10 children. More than two-thirds of children missed their grandparents and extended family. The loss of face to face school and sporting activities was also significant for many children.

Given that their children were spending more time on their own in their room and using technology more, a substantial portion of parents were concerned about the safety of children online. A quarter of the parents surveyed were worried about how to best protect their children from online bullying. A third of parents were worried about how to keep their children from being abused or exploited when they are using the internet. They feel ill equipped to know how to manage.

In the face of problems that are urgent and the subject of government, institutional and community responses (such as COVID-19, crime, economy, environment), child abuse languishes outside the consciousness of the vast majority of the population. 7 in 10 of respondents could not remember seeing or hearing anything about child abuse in the media in the past 12 months. This was even more surprising given the ongoing media reports in the aftermath of the Royal Commission into Institutional Responses to Child Sexual Abuse in Australia—a national formal inquiry which captured the public attention about the ways that children were systematically abused and exploited for decades within religious, sporting, out of home care and other organizations (Tucci and Blom, 2019).

Interestingly, 6% of respondents were not worried about anything at all in their community. This confirms the results of previous studies that the community must be reminded of child abuse before any attention is paid to it.

#### The community is grossly uninformed about child abuse despite believing the issue to be well understood

The results presented in Table 2 show that, as a community, it is not surprising that adults want to feel that they understand such a critical issue as child abuse. Of course, people want children to be safe. This is reflected in the majority of respondents (70%) believing that child abuse was more than adequately recognized as a serious community problem. However, there appears to be a profound disconnect between what the community thinks it knows and what it actually does know about the true size and extent of child abuse as a community problem.

**TABLE 2:** Key findings.

7 in 10 respondents believed that child abuse was fairly well or very well recognized as a serious community problem.
However, 56% were so poorly informed that they could not even hazard a guess at the number of reports of child abuse received last year in Australia.
Of those willing to hazard a guess, almost all grossly under-estimated the number of reports, suggesting less than 10,000 reports were received in 2019–20 when the actual number was over 480,000.
There is a lack of knowledge and confusion about which form of abuse occurs most frequently in Australia.
When asked directly, 86% of respondents argued that the community still needs to better understand the extent and nature of child abuse in Australia.

When asked to estimate the number of reports of child abuse made each year to child protection authorities 56% were so poorly informed that they were unwilling to even hazard any sort of guess. Of the remaining 44% who were willing to give an answer, the vast majority (35%) perceived the number to be less than 10,000—a small fraction of the real figure.

According to the Australian Institute of Health and Welfare, there were 486, 300 notifications of child abuse in 2019–20.

If the more conservative figure is used representing the total number of reports of child abuse that led to a direct investigation by child protective services, then the correct number was 183, 300. In this instance only 3% of respondents in the survey were anywhere close to providing an accurate estimate.

Similarly, when asked to identify which forms of child abuse occurred most frequently in Australia, respondents identified sexual abuse and physical abuse to occur the most frequently with emotional abuse and neglect being the forms of abuse to occur least frequently.

In reality, the opposite is true. According to the AIHW (2021), emotional abuse (54%) was the most common type of abuse or neglect substantiated through investigations in 2019–20. This was followed by neglect (22%), physical abuse (14%), and sexual abuse (9%). This misconception is likely influenced by the media being a primary source of information for the community on the issue of child abuse. Media interest is more likely to report cases of serious physical and sexual violence toward children than other forms of abuse or neglect.

#### Children are still not trusted to tell the truth, leaving them in danger

Despite the overwhelming majority of respondents (85%) knowing the harmful implications of not believing a child’s disclosures of abuse, this findings as reported here confirmed previous findings that two- thirds (67%) of respondents believe that children make up stories about being abused or are uncertain whether to believe children when they disclosed being abused. This remains a devastating result for children. It means that children really only have a 1 in 3 chance of finding an adult who will believe them if they tell them that they are being abused or violated. It is far more likely that children will not be believed or in fact perceived as lying.

Critically, 3 in 4 respondents seemed to understand that the experience of abuse was so compromising for children that they were not likely to disclose they were being hurt. Only 1 in 4 respondents believed that children will usually tell someone if they are being abused. With an understanding of how difficult it is to disclose abuse for children, it would appear to be even more important to believe children are telling the truth when they report to an adult.

Respondents understand both how difficult it is for children to disclose abuse and how devastating it can be for children to be perceived as not telling the truth and yet many continue to hold the view that children cannot be trusted.

These results provide an invaluable insight into why it is not surprising that the Royal Commission into Institutional Responses to Child Sexual Abuse (2017) found that

*“…Of survivors who told us about barriers to disclosure during their private session, more than one in five (22.6 per cent) who said they had disclosed as an adult and more than a quarter (26.1 per cent) who told us they disclosed in childhood said they had thought they would not be believed…”*

Nor, that they also reported that

*“….Many victims do not disclose child sexual abuse until many years after the abuse occurred, often when they are well into adulthood. Survivors who spoke with us during a private session took, on average, 23.9 years to tell someone about the abuse and men often took longer to disclose than women (the average for females was 20.6 years and for males was 25.6 years)…(Royal Commission into Institutional Responses to Child Sexual Abuse, 2017)”*

These results are replicated around the world. Child USA (2020) (a think tank on child protection) also found that

*“…While it may seem intuitive that a survivor would disclose abuse when it happened, data reveals a different reality. In a study of over 1,000 survivors, the average age at the time of reporting child sex abuse was about 52 years….”*

Children continue to face many barriers that prevent disclosure. They often lack the knowledge needed to recognize and understand abuse, lack the ability and language to articulate that they have been abused, do not have an adult they can disclose their abuse to, do not have opportunities to disclose abuse, and ultimately are not believed when they try to disclose. Most disclosures fail to reach individuals who can report the situation and stop the perpetrator. Research shows that, when child victims do disclose, a large percentage of the disclosures are to peers instead of parents or authority figures.

Brattfjell and Flam (2019) have argued that disclosures are more a process than a single event involving

*“…telling through direct and indirect hints and signs, decisions to tell, indecision and delaying, or withholding until adulthood, the dependency on trusted confidants who ask and listen for final disclosure to occur….”*

Rather than occurring in a single moment, the process of disclosure means that the truth can take decades to finally emerge. The experience for adult survivors of abuse often replicates their experience as children. They are asked questions which cast doubt on their story. They are interrogated as to why it has taken so long to come forward. They are threatened and their integrity is impugned.

After almost two decades in which there has been no shift in the prevailing attitude that children lie about their experience of abuse, it is time for a concerted community effort to change this collective mindset and trust children’s truth about their own violation.

#### Children are blamed for the behavior of abusive adults

There remain many pervasive beliefs that form the basis for dismissing or minimizing the true scale and impact of child abuse (Table 3). A small but significant proportion of respondents believed that children are responsible for their own abuse. It reflects the continued victim blaming of children and young people in relation to their experiences of violence. For example, it is akin to the damaging cultural myth that women who “dress or behave provocatively are asking to be assaulted” (CASA FORUM, 2014). In what caused a furore at the time, the then Governor General of Australia, Dr. Peter Hollingworth publicly blamed a 15 year old young woman for being sexually exploited claiming that it was “not sex abuse” by a priest, but “rather the other way round” (Robertson, 2020).

**TABLE 3:** Key findings.

1 in 6 respondents believed that sometimes children are responsible for the abuse they receive from others.
1 in 6 respondents believe that an adult should not be blamed for abusing a child if they get so angry that they lose control.
14% of respondents were uncertain or did not believe that parents who have physically abused and caused injuries to their child should be charged by the police.
11% of respondents were uncertain or did not believe that a parent who punches a child is committing physical abuse.

This belief shifts responsibility away from the perpetrator of the violence and onto the victim. It is further reinforced by the finding that 1 in 6 of the respondents believed that adults should not be blamed for abusing a child if they get so angry that they lose control. In these circumstances, the adult’s behavior is positioned as normal and legitimate—something that everyone can understand and hence condone. Similarly, 14% of respondents did not believe that parents should be held accountable if they physically assaulted and caused injuries to their child. A further 11% of respondents were uncertain or did not believe that a parent who punches a child is committing physical abuse. It is clear from these findings that children are afforded less protection from violence than adults. An adult who punched another adult would been deemed to have committed an assault. Indeed, the devastating consequences of “one punch” attacks have been the subject of significant community outcry and widely reported on in the media.

There is still an unwillingness to re-examine the personal behavior of adults. Despite significant support for adults to recognize that their children’s safety relies on them, a strong undercurrent of discriminating against victims and responsibility shifting still exists.

#### Child abuse still happens in someone else’s neighborhood

Echoing results from the previous three studies, there is still confusion about the characteristics of the perpetrators of child abuse (Table 4). This misunderstanding speaks directly to long held myths associated with child abuse. In particular, that child abuse only occurs in poor households with uneducated parents. There is still a belief that children are most commonly abused by strangers rather than individuals known to the child and more than likely a member of his/her family.

**TABLE 4:** Key findings.

Almost 1 in 5 respondents were believed that children were abused by strangers rather than people known to them.
13% of respondents believed that child abuse only happens in poor or disadvantaged families.
3 in 10 respondents did not believe that child abuse is a social problem of direct concern to them.
62% of respondents were worried about the possibility of their children being abused by someone they don’t know.
69% of respondents were worried about the possibility of their children being abused and exploited online.

Continuing to believe in the myth of stranger danger and the view that child abuse occurs as a result of poverty reinforces the community’s tendency to locate the problem outside of families like their own, in neighborhoods that are different to their own. In so doing, it facilitates a harmful collective perception that reduces the urgency to protect children or take personal responsibility to do anything about it. Clearly, almost 1 in 3 of respondents did not believe that child abuse is a problem which affects them directly. This theme is replicated across a range of findings in the analysis section of this article. However, messaging about the risks to children of exploitation online appear to be resonating with the community.

#### More people than ever before turn away from the reality of child abuse

The community is overwhelmed by the issue of child abuse. It is disheartening, confronting and stressful for many. It reflects the reality of the ongoing threat and danger that face children and young people every day. With such intensity involved in the reaction to child abuse for adults, it is no wonder that they prefer to turn away from it and to an extent deny the seriousness of its scale and effects for children, families and the community more broadly.

#### Reluctance to act leaves children unprotected

As noted in, a small but significant proportion of adults are reluctant to take action to protect children from being abused even if they were certain of the facts. Children require adults to act protectively in order for them to be safe from abuse. Adults in the community are the early responders for children who are at risk of being abused. Yet, if these responders do not believe children or fail to take action, children remain without the backup they urgently require. Messaging from many governments across Australia that child abuse is everybody’s responsibility are falling short.

#### Lack of confidence is a key obstacle in protecting children

Many respondents identified their own lack of confidence in recognizing the signs of abuse and knowing what they needed to do to take action to protect children. This lack of confidence has not changed at all in the past decade with almost identical results being identified in the 2009 study. Knowledge, confidence and skills are core elements of community capability.

Without these qualities, the community is not able to stand up for children, leaving them arguably in danger.

#### Long standing barriers to taking action to protect children from abuse continue to exist

As highlighted by the findings in Table 5, there are persistent barriers acting to restrain individuals from taking action to protect children. Getting it wrong and falsely accusing parents of abuse is at the top of this list. A lack of confidence about what to do was identified again in this list. Fear for their own safety if they take action is significant. However, some are rhetorical beliefs that can be used to justify a lack of action. For example, believing that authorities will not be able to help or not wanting to get involved represent a different kind of barrier which reflects attitudinal positioning aimed at softening the unwillingness of the individual to not follow through with the information they have. This is not uncommon, there are broader discursive themes which are implicated in this lack of action, such as the sanctity of the family unit, the dissonance between the individual and society ownership of social problems, the myth that if it is serious enough someone else will take action.

**TABLE 5:** Barriers to taking action to protect a child known to be abuse.

I may feel unsure the abuse was actually taking place	30%
I may not know what the right thing to do is	21%
I don’t know who to contact to help abused or neglected children	9%
I worry that I might make a false allegation of abuse	32%
I may feel it was not my responsibility to do something	11%
I may not want to get involved	16%
I may have fears for my own safety if I do something	24%
I may be worried the family involved might be broken up	17%
I don’t think the authorities would be able to help	11%
None	14%
Don’t know/No answer	7%

There is the need to actively address each of these barriers with community education. Without concerted effort to change, it is likely that these barriers will continue as they have for at least the last two decades.

#### There is common agreement about the categories of abuse and neglect which warrant further action

When faced with some scenarios there appears to be general consensus about them constituting abuse or neglect (Table 6). This is important because the threshold to have child protection to become involved and investigate reports or offer support to children is a contentious debate. The threshold itself is never articulated or defined. It is often reported that community standards differ according to range of factors, including cultural background of reporters, their qualifications, their experience in reporting previously.

**TABLE 6:** Key findings.

81% of respondents believed that a 4 year old child wandering the streets unsupervised is a form of neglect.
78% of respondents believed that a child who knocks on your door asking for food, saying there is no food in their house and they are hungry is suffering from neglect.
91% of respondents believed that a teacher who texts a 14 year old asking him/her to meet to have sex is sexual abuse or grooming.
79% of respondents believed that a child who goes to school regularly without lunch is being neglected.
64% of respondents believed that a parent who regularly leaves an 11 year old to look after a 6 year old is being neglectful.
79% of respondents believed that a child being cared for by a parent who has a serious drug habit is at risk of neglect.
80% of respondents believed that an 8 year old being locked outside the house for 1 h as punishment is at risk of neglect or emotional abuse.
74% of respondents believed that a baby regularly left to cry for more than an hour at a time is at risk of neglect or emotional abuse.
72% of respondents believed that a parent constantly yells at a child is causing emotional or psychological abuse.

However, these results also suggest that there is greater consensus for some scenarios than others. The exercise of seeking feedback from the community about what constitutes abuse and neglect is a potential innovation that can be used by child protection authorities to determine the circumstances when they should become involved.

#### A significant proportion of adults continue to not recognize significant acts child abuse and neglect

There is virtually no change in the number of respondents who had difficulty in recognizing clear examples of at risk or abusive situations for children. The lack of consensus on these sorts of adverse childhood experiences represents a significant barrier to taking action to protect children in these sorts of circumstances (Table 7).

**TABLE 7:** Key findings.

12% of respondents were uncertain or did not believe that a 14 year old having sex with a 25 year old adult is sexual abuse.
28% of respondents were uncertain or did not believe that 15 year old having sex with an 18 year old adult is sexual abuse.
10% of respondents were uncertain or did not believe that a child or teenager who is manipulated into sending a naked or semi-naked photo of themselves to an adult is being subject to grooming or sexual abuse/exploitation.
12% of respondents were uncertain or did not believe that a parent who downloads photos and videos of children being sexually abused is a form of child abuse or exploitation.
11% of respondents were uncertain or did not believe a public transport employee who secretly records or photographs up children and teenagers’ dresses was a form of sexual abuse.
19% of respondents were uncertain or did not believe a 4 year old child wandering the streets unsupervised is a form of neglect.

#### A significant number of people have identified child abuse and neglect in the past 5 years

In 2009, 26% of respondents had identified a child or young person who had been abused or neglected in the past 5 years. The findings in Table 8 suggest even more people are identifying abuse. In the earlier survey it was not possible to undertake a detailed analysis of the kind of violation to which children had been subjected. In this study, a new set of questions were asked to specifically understand the nature of the abuse that respondents had identified.

**TABLE 8:** Key findings.

38% of respondents had witnessed a child or teenager being humiliated or criticized by an adult family member over the past 5 years.
22% of respondents had witnessed a child or teenager being physically abused by an adult family member over the past 5 years.
23% of respondents had heard someone make sexually suggestive comments or jokes about a child or teenager over the past 5 years.
18% of respondents had had a child or teenager disclosed that they were being abused or hurt by an adult over the past 5 years.
30% of respondents knew of a child or teenager who was living with family violence at home over the past 5 years.
30% of respondents suspected a child or teenager was experiencing abuse over the past 5 years.
18% of respondents knew of a child or teenager who had experienced sexual abuse or exploitation online over the past 5 years.

The results in Table 8 suggest that there are significant numbers of incidents of child abuse and neglect that respondents have come across in the course of their daily lives.

#### Many people feel sorrow, anger and powerlessness when they come face to face with child abuse

The initial reactions of respondents who identified children who had been abused or neglected are listed in the above table. These offer a more detailed insight into the drivers of adult behavior in relation to taking action to protect vulnerable and at risk children.

The findings presented in Table 9 paint a picture of the anger, shock, sorrow, frustration, and powerlessness experienced by adults who become aware a child is being abused. In many ways these feelings mirror the experiences of the very children who are suffering the abuse and neglect. Clearly, there is a need to empower the community in relation to taking action when they become aware that a child is being abused rather than them continuing to feel impotent and a hostage to the problem.

**TABLE 9:** Reaction to the becoming aware of the problem for the child.

Uncertain about what to do	13%
Shocked	13%
Sorry for the child	18%
Angry about the situation	24%
Frustrated I was unable to help	18%
Guilty for not helping	8%
Don’t know/No answer	7%
Total	100%

#### People are willing to act if resourced and supported to do so

The action that each adult took after identifying the abuse and neglect is described in Table 10. In this question, respondents may have indicated that they took more than one action.

**TABLE 10:** Action taken by respondents in response to their concerns.

Discussed my concerns with a family member/friend to get their advice	30%
Talked to the child who was the subject of the concerned	26%
Discussed my concerns with a professional (e.g., teacher, doctor, social worker)	22%
Talked to the person who was harming the child	17%
Reported concerns to child protection authorities	16%
Reported concerns to the police	14%
Phoned a helpline for advice	14%
Did nothing	17%
Other	6%

Of most concern is the 1 in 6 that did nothing to protect children they were worried about. This leaves many children in real danger.

Importantly, the results also showed that 83% took some form of action. 30% of respondents took direct action that could have led to the protection of the child by reporting it to statutory child protection authorities and/or the police. Other responses were less direct and involved seeking advice from trusted others in the community or discussing concerns with the parent. Surprisingly, 1 in 4 of respondents took the step of talking about the concerns directly with the child, possibly before deciding what to do next. 1 in 6 raised the issue directly with the person who was suspected of being the perpetrator of the abuse. With nearly one third of respondents talking to trusted people within their own informal networks, the need to equip the community with knowledge and empower them to take action is again demonstrated in these findings.

#### When driven to act, it occurs quickly

The time taken for individuals to take action is set out in Table 11. Of those who took any action, almost a third responded immediately. A further 32% responded within a week. 1 in 10 (11%) took more than a month and some over a year. These results highlight that individuals who are motivated to take action will do so quickly and decisively.

**TABLE 11:** Period of time before respondent took action after being concerned about the child.

Same day	34%
Within a week	32%
Within a month	14%
More than a month but less than 6 months	5%
Between 6 and12 months	1%
1 year or more	5%
Total	100%

#### A sense of responsibility and concern drives action for many

The main motivation for taking action is listed in Table 12. In this question, respondents may have indicated more than one reason for taking action.

**TABLE 12:** Main reason for taking action.

I acted on my gut instinct and knew I had to do something	25%
I felt it was my personal responsibility to do something	28%
I didn’t think anyone else would take action	16%
I thought the situation was serious and needed immediate action	19%
It’s part of my job to protect children	16%
I didn’t want to have regrets later about not doing something at the time	20%
I cared about the child concerned	35%
I was worried about the long-term consequences for the child if I didn’t do something	33%
I thought the family was under stress and needed help	14%
Don’t know/No answer	4%

Individuals engaged with their own commitment to the child or their social responsibility, as adults, to protect children. Some saw that their action would lead to the whole family receiving assistance. For others, it was the thought that they had to act because they were the last resort for the child in question. A small proportion of respondents were compelled to act as a way of avoiding feeling regret later if the child continued to be harmed.

## Taking action helps children

As noted in Table 13, for those that did take action, over half (55%) believed their intervention resulted in improved safety for the child. Smaller proportions of respondents did not know about the impact of their actions or believed that the safety of the child has been further compromised by their involvement.

**TABLE 13:** Outcomes of action taken by respondents.

Made things much better	22%
Made things a little better	33%
Made no difference at all	15%
Made things worse	5%
Don’t know if it made a difference	25%
Total	100%

### Confusion and uncertainty stops people taking action

1 in 6 (17%) of respondents stated they took no action at all. The main reasons for not taking action, despite being concerned about the possibility of a child being abused, are described in Table 14. In this question, respondents may have indicated more than one reason for not taking action.

**TABLE 14:** Main reason for not taking action.

I was unsure the abuse was actually taking place	24%
I didn’t know what was the right thing to do	22%
I didn’t know who to contact to help the child	6%
I was worried that I might make a false allegation of abuse	25%
I didn’t think it was my responsibility to do something	5%
I didn’t want to get involved	24%
I had fears for my own safety if I did something	17%
I was worried the family involved might be broken up	15%
I didn’t think the authorities would be able to help	3%
Someone I spoke to about the situation advised me not to do anything further	6%
No, none	5%
Other	19%
Don’t know/No answer	9%

A quarter of the respondents who did not take action were uncertain about whether or not the abuse was actually taking place. A much smaller proportion (6%) followed the advice of another person to take no action. A significant proportion (17%) identified legitimate concerns about their personal safety as a reason for not taking action.

However, the remaining reasons for not taking action reflected a number of critical barriers that are derived from an active avoidance of the problem of child abuse. These include not wanting to become involved, not knowing what steps to take and fearing that intervention would make the situation worse for the child.

### There is significant impetus to prioritize the prevention of child abuse and the protection of children

As noted in Table 15, there is strong recognition that inadequate investment in strategies to reduce the extent of child abuse will lead to severe consequences for the community. It follows that there is also a high degree of interest to be better informed and more actively involved in efforts to prevent child abuse.

**TABLE 15:** Key findings.

75% of respondents believed that there is a need for national campaigns to raise awareness of child abuse and the need to protect children from child abuse.
45% of respondents would be prepared to become actively involved to support a campaign or event(s) that helped the community know how to recognize child abuse and be more confident to act.
85% of respondents believed that if we do not prevent child abuse now, the long term consequences for the community are enormous.
80% of respondents argued that more money should be invested in protecting children from child abuse and neglect.

## Discussion

### Child abuse remains largely unseen and ignored

Birrell and Birrell (1966) had to fight community disbelief and professional skepticism to raise public alarm about the impact and scale of child abuse in Australia.

In a follow up to their original paper, Birrell and Birrell (1968) wrote that it was clear that

*“…our community, despite some understanding of the problem, still has a long distance to travel in the recognition of this problem…(p. 1028).”*

Over five decades later, the results of the current study suggest that there is still a gulf between the reality of child abuse as a societal problem and sufficient community appreciation of it.

In September 2003, we released the first results of a national community attitude tracking study about child abuse and child protection (Tucci et al., 2003). At that time, we argued that as a community, violence against children was tolerated. The community did not understand or appreciate the seriousness, size and cost of child abuse in Australia. There was evidence that child abuse was not viewed as an important challenge facing children in Australia. A second study conducted in 2006 (Tucci et al., 2006) found that nothing much had changed, indeed community engagement with the issue of child abuse may have even deteriorated. A third study in 2010 found that the community actively avoids the problem of child abuse rating it less concerning than high petrol prices.

In 2021, 18 years after the first report was published, we have concluded again that child abuse remains largely unseen and ignored as a community concern. The results are virtually identical to those found over the past three earlier studies. In 2021, child abuse rates lower than problems with public transport and roads on a list of community concerns. In 2021, 7 in 10 of respondents could not remember seeing or hearing anything about child abuse in the media in the past 12 months.

In 2006, 43% respondents felt so poorly informed on the issue so as to be unable to guess at the number of reported cases of child abuse, whilst those prepared to estimate, significantly underestimated the problem. In 2021, 56% were so poorly informed that they could not even hazard a guess at the number of reports of child abuse were received last year in Australia. This is an 13% increase over that time.

In 2003, the community was extremely ambivalent about trusting children. Thirty-five (35%) percent of respondents would not believe children’s stories about being abused. In 2006, 31% of respondents stated that they would not believe children’s stories about being abused. In 2021, 32% of respondents believed that children can make up stories about being abused.

In 2003, just over 1 in 3 respondents did not believe that child abuse was a problem that they needed to be personally concerned about. In 2021, the result was exactly the same.

In 2010, 1 in 6 of respondents did nothing when faced with a child they believed was being abused. In 2021, the result was exactly the same.

In 2006, additional concerns came to light for the first time. For example, 1 in 5 of respondents in the survey lacked the confidence to know what to do if they suspected that a child was being abused. In 2021, 1 in 5 (22%) were not confident about knowing what to do if they suspected that a child was being abused or neglected. In addition, 1 in 4 (27%) were not confident of being able to recognize that a child was being abused or neglected.

The community lacks all of the building blocks required to prevent child abuse and adequately act to protect them from abuse and neglect. They are not aware of the true scale and impact of child abuse. They do not believe that it is as widespread as it really is. They have a shallow understanding of how it is defined, what its components are, how it develops or the level of risk that children and young people face in their own homes. They lack confidence about when, what and why they should take action when exposed to information that children are being abused and neglected. There are still prevailing attitudes that stop them from stepping up to keep children safe. These attitudes have been there for at least 18 years and they have not changed.

### Children are left unprotected

There is still significant proportion of adults who do not perceive that taking action to protect children from abuse is their responsibility. They continue to be influenced by powerful and inaccurate myths and beliefs such as

• children lie when they disclose abuse;
• child abuse only happens in poor or disadvantaged families;
• outsiders should not interfere into the private lives of families; and,
• children are to blame for the abusive behavior of adults and are somehow therefore less deserving of our protection.

These mindsets shape the behavior of many adults. It makes them more susceptible to perceiving why they should not take action to protect children. For example, respondents who had become aware of a child who was being abused in the past 5 years identified not knowing the right thing to do, being worried that they would be accused of making a false allegation and not carrying any responsibility to act as key reasons for doing nothing.

Such biases are inherently connected to broader themes that are reinforced by the reporting in the media (FrameWorks Institute, 2003, 2004; FrameWorks Institute, 2009, 2015). These include the perception that

• children will always be abused, it is part of human nature;
• systems are not working so there is little we can do that will make a difference;
• there is no sense of community anymore, so why should we bother, the best I can do is to look after me and my loved ones;
• child abuse does not touch my life directly, I do not need to be worried about it; and,
• perpetrators are really cunning, they have been getting away with abusing children for years, not even the police can stop them.

Each of these examples highlight how disempowering prevailing narratives are for adults who may be motivated to act in the best interests of children but end up being overwhelmed by the sheer weight of obstacles that they perceive to be in their way.

At every turn, each of these themes increases the uncertainty that adults experience as they determine how to evaluate the information they have about a child and ultimately how they choose to act. The greater the uncertainty, the greater the likelihood of inertia and in turn the higher the likelihood that children are left unprotected.

It is only when adults engage with their sense of social responsibility that they act. This is a finding that has been replicated elsewhere (FrameWorks Institute, 2004, 2009, 2015; NAPCAN, 2010). In this study, respondents cited the following reasons as being the main motivations behind their decision to actively intervene to protect a child they knew was being abused or neglected:

• I knew I had to do something;
• I felt it was my personal responsibility to do something;
• I didn’t think anyone else would take action;
• It’s part of my job to protect children; and,
• I didn’t want to have regrets later about not doing something at the time.

These are the clearest results to date in favor of a strong and detailed community education campaign that builds the case for why, how and when adults need to act to keep children safe from abuse.

### The community is turning away and ill-informed

Over the past decade, it appears the community is finding it more and more difficult to face up to the reality of child abuse with increasing numbers reporting they find talking about child abuse tense and difficult and that they cannot bear to see images of children who have been hurt or neglected.

In this survey, 44% of respondents reported feeling tense and anxious when they take part in a conversation about child abuse. This is an increase of 16% since 2010 when the last study in this series was undertaken. In addition, 71% of respondents reported that they cannot bear to look at pictures of children in the media who have been hurt or neglected. This is an increase of 12% since 2010 when the last study in this series was undertaken.

Perhaps due to ongoing stress directly arising from COVID-19 and the fatigue of the ongoing consequences for the community broadly (Tucci et al., 2020), more people than ever before find it hard to stay engaged with the intensity of the reality faced by so many children who are being abused or neglected. It is as if when there is community wide danger, the risks to children need to be pushed even further away from individual and community awareness. It is a threat that is just too much to handle. It acts to make the world feel so much vulnerable at a time when uncertainty is so prevalent.

The end result is that individuals turn away from the reality of child abuse because they find the pain suffered by children intolerable. It is inevitable that a problem that the community is forced to hide from is a problem that stays in the shadows and away from active engagement and efforts to resolve. Looking away is easier than looking into the eyes of children who have been hurt and traumatized by the very adults who are supposed to care and nurture them.

All social movements that result in collective and effective common action commence with the realization of the crisis that is occurring and the way that such escalating problems affects each person in the community. Concerted action about the environment has required the collaboration of different sectors of the community playing a role to prove the existential threat it represented to the current and future generations. It requires uncomfortable truths to be realized and accepted. This is still not the case for child abuse. Its long term ramifications have been proven by the weight of scientific evidence (Tucci et al., 2019).

The cost to the community has been estimated in the billions of dollars (Taylor et al., 2008; McCarthy et al., 2016). It is at the core of downstream social consequences such as poor health, unemployment, mental illness, addiction, suicide, and more.

Yet, despite efforts to the contrary, child abuse appears to remain, at its most basic level, a topic that sits on the periphery of community consciousness.

Clearly, the results of this survey show that many people feel sorrow, anger, and powerlessness when they come face to face with child abuse in their own families and communities. They are shocked, feel sorry for the child, experience anger that children are being hurt and frustrated or guilty at not being able to help the child.

The act of turning away from it prevents the community from learning what it needs to know in order to be empowered enough to act to prevent it in the first place. The results demonstrated that 9 out of 10 people acknowledge that in its effort to buffer the pain that children suffer, the community stays uninformed about the real extent and nature of the problem of child abuse in Australia.

### The community want and are prepared to do more

There is hope still in these results. Three quarters of respondents supported the need for a national campaign to raise awareness of child abuse and how the community can act more protectively toward children. Just under half of respondents would be prepared to become actively involved to support a campaign that helped the community know how to recognize child abuse and be more confident to act. Over 8 out of 10 respondents believed that if there was inadequate action taken to prevent child abuse now, the long term consequences for the community are enormous.

### Changing the story

For almost 40 years, raising public awareness of child abuse through mass media campaigns has been widely recognized as an effective primary prevention strategy (Daro, 1988; Daro and Gelles, 1992; New South Wales Child Protection Council, 1995; Jernigan and Wright, 1996).

King (1997), for example, suggested that public awareness campaigns be focussed on promoting debate about the notions of childhood which provide an opportunity for society to consider what is and is not in the interests of children. Rayner (1995) argued that the prevention of child abuse was predicated on creating and maintaining a “non-abusive” society and a healthy family environment in which children’s rights to safety and security are respected and optimized. Similarly, the New South Wales Child Protection Council (1997) proposed that the promotion of a “child friendly society” was the cornerstone of the prevention of child abuse.

The need for public awareness and community education campaigns to tackle abuse and neglect of children in Australia is well documented with significant consensus on the need for these programs to be multi-faceted and utilize a range of communication strategies (National Child Protection Council, 1995; Tomison and McGurk, 1996; New South Wales Child Protection Council, 1997; Tucci et al., 1998, 2001, 2003, 2006, 2010; Repucci et al., 1999).

We need to support education with national legislation. Current laws in relation to child abuse differ markedly from state to state in Australia. These differences contribute to the confusion about how to define, identify and respond to child abuse.

Community attitudes can be changed if the public has access to a clear and unequivocal framework for understanding the issue of child abuse. We need national uniform laws which set out standards for defining, identifying, reporting and investigating cases of child abuse, family violence and neglect.

That is why all levels of government need to commit to resourcing sustained public education campaigns aimed at engaging the community in the protection of children from abuse. Increasingly, there has been significant public investment in the past 5 years in using community education campaigns to address gender inequality as the upstream factor leading to family violence (Our Watch, 2022). This has not translated into similar resourcing of campaigns in relation to child abuse prevention.

Importantly, State and Commonwealth Governments need to urgently co-operate to develop and implement uniform national child abuse and child protection legislation.

And finally, individuals need to find within themselves the commitment to listen to and believe children, especially in relation to child abuse and family violence.

## Conclusion

*“…statistics paint a horrendous picture of an Australian childhood stolen by trauma, abuse and violence. It is unquestionably one of the most pressing and critical social problems…(Tucci et al., 2006).”*

In 2021, child abuse remains unseen and largely ignored in particular in the face of so many other issues facing the community. As this survey was conducted during a worldwide pandemic, so were earlier studies conducted at times of significant worldwide and national problems, such as the risks of terrorism.

Children cannot afford competing demands for community attention to detract from their fundamental entitlements to safety, love and care. The reality of the other challenges confronting the community is not a reason to do nothing. The most vulnerable and at risk children cannot be left to wait whilst larger problems are addressed. The problem does not go away if we choose to turn away from it. Difficult challenges facing the community require strong leadership, an understanding of where the community is up to in its understanding and what it needs to be more empowered. The results of this survey have again mapped the challenges faced by vulnerable, frightened and unprotected children and young people in the community. They have not changed, if anything the problems they experience are further compounded.

## Data availability statement

The raw data supporting the conclusions of this article will be made available by the authors, without undue reservation.

## Ethics statement

Ethical review and approval was not required for the study on human participants in accordance with the local legislation and institutional requirements. The patients/participants provided their written informed consent to participate in this study.

## Author contributions

Both authors listed have made a substantial, direct, and intellectual contribution to the work, and approved it for publication.
