# Supplementary material for: Still unseen and ignored: Tracking community knowledge and attitudes about child abuse and child protection in Australia
Source: Front Psychol. 2022 Sep 2;13:860212. doi: 10.3389/fpsyg.2022.860212 (PMC9480499; doi:10.3389/fpsyg.2022.860212)
Supplement: Supplementary file 1 [file Data_Sheet_1.docx]

# Appendix A - Survey Sample

| **Table A.1 Age demographics of sample** |  |  |
| --- | --- | --- |
| 18 to 24 | 12% | 88 |
| 25 to 34 | 19% | 188 |
| 35 to 44 | 17% | 183 |
| 45 to 54 | 16% | 168 |
| 55 to 64 | 15% | 159 |
| 65+ | 20% | 223 |
| **Total** | **100%** | **1009** |

| **Table A.2 Gender demographics of sample** |  |  |
| --- | --- | --- |
| Male | 49% | 477 |
| Female | 51% | 532 |
| Gender diverse | 0% | 0 |
| Prefer not to say | 0% | 0 |
| **Total** | **100%** | **1009** |

| **Table A.3 Geographical spread of sample** |  |  |
| --- | --- | --- |
| NSW | 32% | 326 |
| VIC | 26% | 278 |
| QLD | 20% | 197 |
| WA | 11% | 108 |
| SA | 8% | 65 |
| TAS | 2% | 24 |
| ACT | 1% | 7 |
| NT | 1% | 4 |
| **Total** | **100%** | **1009** |

| **Table A.4 Locality demographics of sample** |  |  |
| --- | --- | --- |
| METRO | 72% | 719 |
| REGIONAL | 28% | 290 |
| **Total** | **100%** | **1009** |

| **Table A.5 Household composition of sample** |  |  |
| --- | --- | --- |
| Households with children | 43% | 429 |
| Households with no children | 57% | 580 |
| **Total** | **100%** | **1009** |

# Appendix B. Spontaneous Recall Findings

| **Table B.1 Issues of most concern (spontaneous recall)** | **2003** | **2006** | **2010** | **2021** |
| --- | --- | --- | --- | --- |
| COVID-19 | - | - | - | 24% |
| Economy | - | - | 9% | 16% |
| Crime | 28% | 24% | 26% | 12% |
| Unemployment | 9% | 5% | 10% | 10% |
| Environment | 14% | 9% | 17% | 7% |
| Not concerned about anything | - | - | - | 6% |
| Health | 19% | 15% | 18% | 5% |
| Transport, Traffic and Roads | 7% | 5% | 11% | 3% |
| Homelessness | - | - | - | 3% |
| Inequality | 4% | 5% | 3% | 2% |
| Mental Health | - | - | - | 2% |
| Drugs | 11% | 7% | 8% | 2% |
| Child abuse | 2% | 4% | 2% | 1% |
| Education | 14% | 12% | 9% | - |
| Terrorism | - | 9% | - | - |
| Aged care | 3% | - | 3% | - |
| Council rates | 3% | - | - | - |
| Asylum seekers | 3% | - | - | - |
